# *Anemarrhena asphodeloides* Polysaccharide Exerts Antiviral Activity Against Porcine Epidemic Diarrhea Virus Targeting ROS/Caspase‐3 Dependent Apoptosis

**DOI:** 10.1155/tbed/9703618

**Published:** 2026-04-21

**Authors:** Long Yin, Xiaotong Wang, Jianbin Wang, Yue Zhang, Wei Zhang, Ping Yan, Jingting Yao, Luyao Jiang, Song Gao, Changchao Huan

**Affiliations:** ^1^ Institute of Agricultural Science and Technology Development, College of Veterinary Medicine, Yangzhou University, Yangzhou, China, yzu.edu.cn; ^2^ Jiangsu Youshi Biotechnology Development Co. Ltd, Suqian, China; ^3^ Jiangsu Co-Innovation Center for Prevention and Control of Important Animal Infectious Diseases and Zoonoses, Yangzhou, China, yzu.edu.cn; ^4^ Key Laboratory of Avian Bioproduct Development, Ministry of Agriculture and Rural Affairs, Yangzhou, China, agri.gov.cn; ^5^ Qingdao Vland Animal Health Group Co., Ltd, Qingdao, China; ^6^ Joint International Research Laboratory of Agriculture and Agri-Product Safety of Ministry of Education of China, Yangzhou University, Yangzhou, China, yzu.edu.cn

**Keywords:** *Anemarrhena asphodeloides* polysaccharide, antiviral, apoptosis, porcine epidemic diarrhea virus, ROS

## Abstract

Porcine epidemic diarrhea (PED) is the severe infectious disease caused by the porcine epidemic diarrhea virus (PEDV), characterized by the onset of watery diarrhea. *Anemarrhena asphodeloides* polysaccharide, extracted from *Anemarrhena asphodeloides*, presents potential applications as immunostimulatory and anticancer agent in alternative therapies. However, its antiviral activity remains unreported. This study investigates the inhibitory effects of *Anemarrhena asphodeloides* polysaccharide on PEDV infection. The findings demonstrate, for the first time, that *Anemarrhena asphodeloides* polysaccharide can effectively inhibit PEDV infection in a dose‐dependent manner. It does not impact PEDV release and deactivation. Through the network pharmacology and bioinformatics analysis, we focused on the antiviral mechanism of *Anemarrhena asphodeloides* polysaccharides in terms of apoptosis and oxidative stress. We revealed that *Anemarrhena asphodeloides* polysaccharide significantly decreases oxidative stress and caspase‐3 dependent apoptosis in infected cells. Apoptosis inhibitor Ac‐DEVD‐CHO and ROS scavenger NAC disrupt PEDV infection, and NAC reduces caspase‐3 dependent apoptosis. This study provides preliminary evidence of the anti‐PEDV activity of *Anemarrhena asphodeloides* polysaccharide by targeting ROS/caspase‐3 dependent apoptosis, establishing it as novel anti‐PEDV agent.

## 1. Introduction

Porcine epidemic diarrhea (PED) is the severe pig disease caused by the coronavirus known for its severe watery diarrhea and weight loss, with high mortality rate among newborn piglets [1, 2]. It emerges sporadically during the winter, resulting in significant livestock farm losses. Although PED shares similarities with transmissible gastroenteritis (TGE), it has lesser impact on suckling pigs [2]. While PEDV circulated in China’s pig population from 1984 to early 2010, large‐scale outbreaks were not observed [3]. However, from late 2010, porcine epidemic diarrhea virus (PEDV) outbreaks occurred in several pig‐producing provinces in Southern China [4], leading to an alarming piglet mortality rate of 80%–100% [5]. The disease caused over a million piglet deaths in Southern China, causing substantial damage to the pig farming industry [6]. PEDV was initially identified in Europe [7] and belongs to the Coronaviridae family as positive‐sense, single‐stranded RNA virus [8]. Given the urgency of PED prevention and control, this study focuses on PEDV treatment using traditional Chinese medicine.

*Anemarrhena asphodeloides* has long history of use in traditional medicine for thousands of years [9]. It is perennial herb that is widely found in China and Korea and has been used as a medicinal plant to treat cough, night sweats, tidal fever, and relieve dryness syndrome [10, 11]. The rhizome of *Anemarrhena asphodeloides* belongs to the Liliaceae family, which possesses antipyretic, hypoglycemic, and immunomodulatory properties [12]. *Anemarrhena asphodeloides* polysaccharide holds potential for use as immunostimulatory and anticancer agent in alternative therapies. It exhibits anticancer effects. However, the impact of *Anemarrhena asphodeloides* polysaccharide on PEDV infection has not yet been explored. This study aims to assess the role and mechanism of *Anemarrhena asphodeloides* polysaccharide in PEDV infection.

## 2. Materials and Methods

### 2.1. Cell Culture and Virus Preservation

Vero cells were derived from the infectious disease laboratory of Yangzhou University. Vero cells were cultured at 37°C with 5% CO_2_, using DMEM containing 6% fetal bovine serum (FBS). PEDV HLJBY is stored at –80°C in the infectious disease laboratory of Yangzhou University.

### 2.2. Chemicals, Reagent, and Antibodies

Polysaccharide (≥98% (UV)) was obtained from Yangling Ciyuan Biotechnology Co., Ltd., China, and was diluted to the concentration of 50 mg/mL with PBS before being stored at –20°C. The PEDV N antibody utilized in this study was saved in our laboratory. Anti‐PEDV N polyclonal antibody was diluted 1:5000 for Western blot and 1:500 for immunofluorescence assay (IFA) [13]. The *β*‐actin antibody was purchased from TransGenBiotech, China. Enzyme‐labeled goat antimouse IgG (H + L) and DAPI (4,6‐diamino‐2‐phenylindole) were obtained from Beyotime Biotechnology, Shanghai, China. The citric acid solution (pH 3.0) was prepared by combining citric acid (40 mM), NaCl (135 mM), and KCl (10 mM) for the removal of uninternalized virus particles.

### 2.3. Infectivity Assay

Vero cells (5 × 10^5^/well) were seeded into 6‐well plates and incubated at 37°C with 5% CO_2_ until they reached the density of 70%–80%. Following the removal of the original medium, the cells were washed three times with PBS. Vero cells were then pretreated with polysaccharide (500 µg/mL) for 1 h. Subsequently, multiplicity of infection (MOI) of 0.1 of PEDV was used to infect the Vero cells for 6, 12, and 24 hpi. For the PEDV infection, PEDV HLJBY (MOI = 0.1) was inoculated into Vero cells after pretreatment with DMEM containing different concentrations of polysaccharide (100, 200, and 500 µg/mL) for 1 h. The cells were then cultured at 37°C in 2% DMEM containing the respective polysaccharide concentrations (100, 200, and 500 µg/mL). At 24 h postinfection, the cells were harvested, and the level of PEDV N protein and PEDV N mRNA were determined by Western blot and qRT‐PCR, respectively. The cell supernatant was collected, and the virus titer was assessed using TCID_50_.

### 2.4. Effects of *Anemarrhena asphodeloides* Polysaccharide on PEDV

PEDV HLJBY (MOI = 0.1) and *Anemarrhena asphodeloides* polysaccharide (100, 200, and 500 µg/mL) were coincubated at 37°C for 1 h. Subsequently, the pretreated virus was introduced into Vero cells and harvested at 24 hpi. Western blot analysis was performed to assess alterations in PEDV N protein.

### 2.5. Target Prediction and Analysis of *Anemarrhena asphodeloides* Polysaccharide and PEDV

The potential targets of PEDV were obtained from GeneCards (https://www.genecards.org/). And, according to previous reports, the active components of *Anemarrhena asphodeloides* polysaccharide include D‐mannose, L‐rhamnose, D‐galacturonic acid, D‐glucose, D‐galactose, and L‐arabinose [14]. Subsequently, the Canonical SMILES expressions of these identified monosaccharides were searched in the PubChem database (https://www.pubchem.ncbi.nlm.nih.gov/). Then, based on the two‐dimensional and three‐dimensional characteristics of the known ligand, potential molecular targets were predicted using the Swiss Target Prediction database (http://www.swisstargetprediction.ch/), and obtained the target genes of *Anemarrhena asphodeloides* polysaccharide by taking the intersection.

### 2.6. Protein–Protein Interaction (PPI), GO, and KEGG Enrichment Analysis

The active compound targets of *Anemarrhena asphodeloides* polysaccharide and PEDV using the Bioinformatics platform (https://www.bioinformatics.com.cn/) to generate Venn diagrams. Overlapping targets were submitted to the STRING database (https://string-db.org/) settings to construct PPI networks.

Gene ontology (GO) enrichment (biological processes [BP], cellular components [CC], and molecular functions [MF]) and KEGG pathway analyses were performed using the DAVID database with the species. The top 10 pathways ranked by *p* values were visualized using the Bioinformatics platform. Finally, the drug‐target‐disease visualization network and drug–target–disease–pathway network were constructed in Cytoscape (v3.10.0).

### 2.7. Intracellular ROS Assay

Vero cells were pretreated at 37°C for 1 h with *Anemarrhena asphodeloides* polysaccharides (100, 200, and 500 µg/mL) and then infected with PEDV HLJBY (MOI = 0.1). After 1 h, the cell supernatant was discarded, and the cells were rinsed three times with PBS. Subsequently, they were cultured at 37°C in 2% DMEM containing *Anemarrhena asphodeloides* polysaccharide (100, 200, and 500 µg/mL). After rinsing the cells three times with PBS at 24 hpi, cells were incubated with a 10 μM solution of 2′,7′‐dichlorofluorescein diacetate (DCFH‐DA) probe for 30 min at 37°C. The excess probe was removed, and the cells were resuspended in PBS. The total fluorescence intensity of more than 10,000 cells in each sample was determined by flow cytometry.

### 2.8. Western Blotting

The collected cells were lysed with cell lysis buffer. The cell lysate protein and 2× SDS‐PAGE sample were mixed with buffer (1:1) and boiled in a water bath at 96°C for 15 min. Proteins separated by 12% SDS‐PAGE were transferred from the gel to a nitrocellulose (NC) membrane. Subsequently, the NC membrane was blocked with 5% skim milk powder in PBST at room temperature for 2 h, then incubated overnight with the primary antibody at 4°C, and washed three times with PBST. The NC membrane was incubated with enzyme‐labeled goat antimouse antibody at room temperature for 2 h. The results of the immune complex band were analyzed using the Super ECL reagent solution kit (Vazyme Biotech Co., Ltd.).

### 2.9. Indirect IFA

Cells were fixed at 37°C for 30 min with 4% paraformaldehyde (PFA), then incubated with 0.1% Triton X‐100 for 10 min, and blocked overnight at 4°C with 5% BSA. After three washes with PBS, cells were incubated at 37°C for 1 h with the anti‐PEDV N antibody and then for 30 min with FITC‐labeled goat antimouse IgG antibody at 37°C. Finally, cells were stained with DAPI for 7 min [13] and observed under the fluorescence microscope.

### 2.10. Virus Titer Assays

Virus titers were assessed by TCID_50_ as previously described [15].

### 2.11. qRT‐PCR

qRT‐PCR was conducted following the previously described protocol [13]. Gene expression was assessed by qRT‐PCR using specific primers listed in Table 1 [16].

**Table 1:** qPCR primer and probe sequences used in this study.

Method	Target	Forward (5′→3′)	Reverse (5′→3′)
TaqMan	PEDV N gene	GAATTCCCAAGGGCGAAAAT	TTTTCGACAAATTCCGCATCT
	N gene probe	FAM‐CGTAGCAGCTTGCTTCGGACCCA‐BHQa	

### 2.12. Statistical Analysis

All experiments were independently repeated at least three times, and the data were presented as the mean ± SD. Data analysis was performed using GraphPad Prism software (GraphPad Software, San Diego, CA, USA) and one‐way analysis of variance (ANOVA). Significant differences were indicated as  ^∗^ *p* < 0.05 ^∗∗^ *p* < 0.01, signifying statistical significance. “ns” was indicated as no significant difference.

## 3. Results

### 3.1. *Anemarrhena asphodeloides* Polysaccharide Inhibited PEDV Infection

The toxicity of *Anemarrhena asphodeloides* polysaccharide on Vero cells was evaluated by measuring cell viability via CCK8 assay after Vero cells were treated with different concentrations of *Anemarrhena asphodeloides* polysaccharide (0, 100, 200, 500, and 1000 µg/mL) for 24 h. The cell activity remained unaltered at concentrations of 100, 200, and 500 μg/mL (Figure 1A).

Figure 1Antiviral activity of *Anemarrhena asphodeloides* polysaccharide. The antiviral activity of *Anemarrhena asphodeloides* polysaccharide was determined as follows: Vero cells were pretreated with different doses of *Anemarrhena asphodeloides* polysaccharide for 1 h before PEDV HLJBY infection (MOI = 0.1). The cells were then cultured in 2% DMEM containing *Anemarrhena asphodeloides* polysaccharide. (A) The cell toxicity of *Anemarrhena asphodeloides* polysaccharide was evaluated by CCK‐8. (B) At 24 hpi, the levels of *β*‐actin and PEDV N protein were determined by Western blot. (C) Virus titer was detected by TCID_50_. (D) Internalized viruses were evaluated by IFA. (E) Quantitative analysis of viral mRNA copy number by qRT‐PCR. (F) Vero cells were treated with 500 μg/mL of *Anemarrhena asphodeloides* polysaccharide for 1 h and then infected with PEDV HLJBY (MOI = 0.1). *β*‐actin and PEDV N protein levels were measured by Western blot at 6, 12, and 24 hpi. The error bar represents the standard deviation of three separate measurements.  ^∗∗^ *p*  < 0.01.

**Figure:**
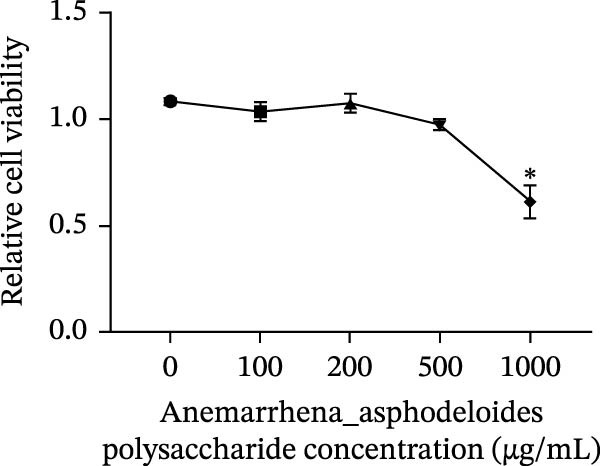
(A)

**Figure:**
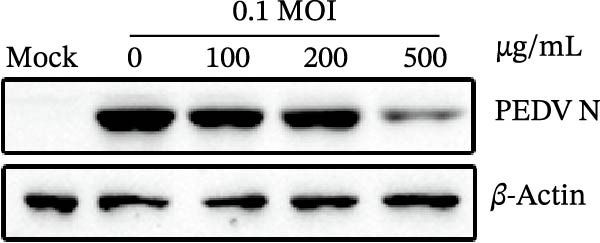
(B)

**Figure:**
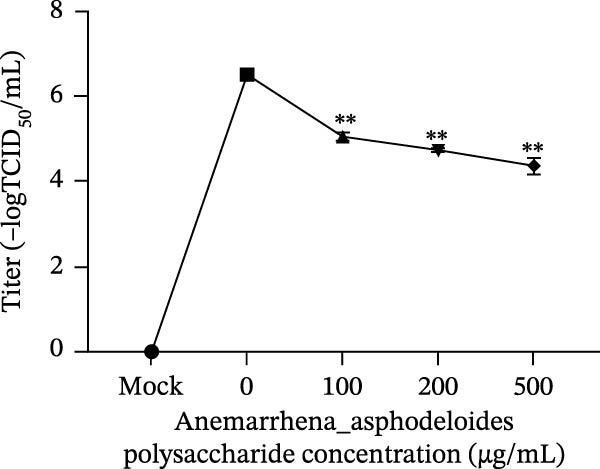
(C)

**Figure:**
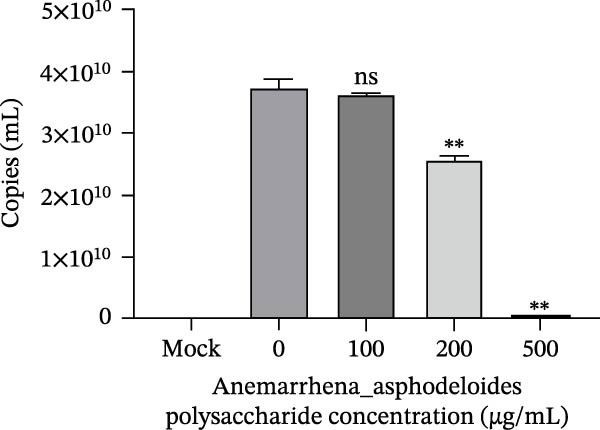
(D)

**Figure:**
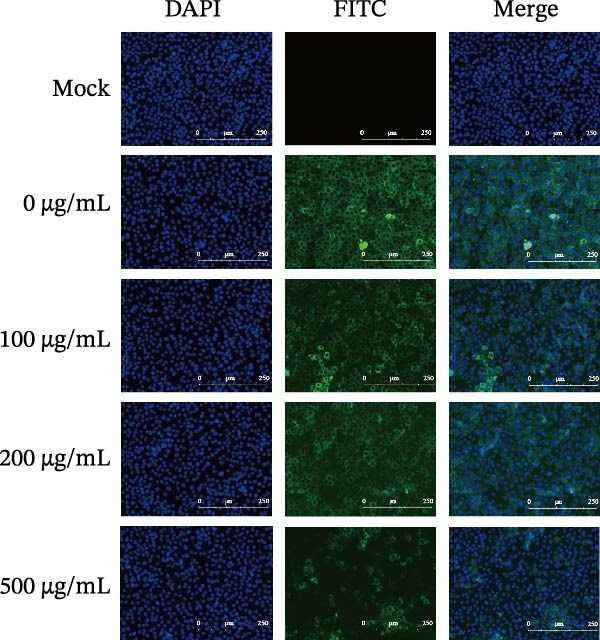
(E)

**Figure:**
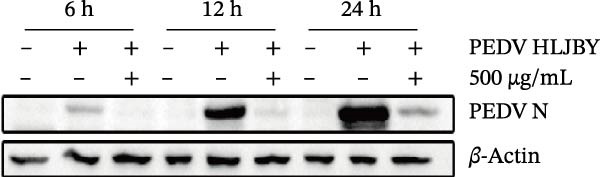
(F)

To assess the antiviral activity of *Anemarrhena asphodeloides* polysaccharide against PEDV infection, Vero cells were treated with different doses of *Anemarrhena asphodeloides* polysaccharide for 1 h. Subsequently, cells were infected with PEDV HLJBY (MOI = 0.1) and *Anemarrhena asphodeloides* polysaccharide for 1 h, followed by three washes with PBS. The cells were then cultured at 37°C with 2% DMEM containing *Anemarrhena asphodeloides* polysaccharide (100, 200, and 500 µg/mL) and collected at 24 hpi. Western blot results revealed the significant decrease in PEDV N protein expression with increasing *Anemarrhena asphodeloides* polysaccharide concentration (Figure 1B). Moreover, virus titers in the cell supernatant were assessed, showing the dose‐dependent reduction in virion production (Figure 1C). Additionally, the number of infected cells decreased with increasing *Anemarrhena asphodeloides* polysaccharide concentration according to IFA results (Figure 1E). qRT‐PCR results demonstrated the dose‐dependent reduction in the copies of PEDV N in the presence of *Anemarrhena asphodeloides* polysaccharide (Figure 1D). These findings indicate that *Anemarrhena asphodeloides* polysaccharide can effectively inhibit PEDV infection in Vero cells. To further explore the antiviral potential of *Anemarrhena asphodeloides* polysaccharide against PEDV, Vero cells were exposed to PEDV HLJBY (MOI = 0.1) along with *Anemarrhena asphodeloides* polysaccharide (500 µg/mL). Cell samples were gathered at 6, 12, and 24 hpi. Western blot analysis revealed the significant inhibitory effects of *Anemarrhena asphodeloides* polysaccharide on PEDV infection at different time points (Figure 1F).

### 3.2. Effects of *Anemarrhena asphodeloides* Polysaccharide on Cells and Virus Inactivation

Vero cells underwent pretreatment with varying concentrations of *Anemarrhena asphodeloides* polysaccharide (100, 200, and 500 µg/mL) and subsequent infection with PEDV HLJBY (MOI = 0.1) for 24 h. Western blot analysis revealed an unaltered level of PEDV N protein (Figure 2A), indicating that cell pretreatment had no impact on virus infectivity. PEDV HLJBY (MOI = 0.1) was pretreated with *Anemarrhena asphodeloides* polysaccharide (100, 200, and 500 µg/mL) for 1 h and then inoculated with Vero cells. Cells were collected at 24 hpi to measure the expression of PEDV N protein. Western blotting analysis revealed no significant changes in PEDV N protein expression, indicating that the *Anemarrhena asphodeloides* polysaccharide could not directly inactivate the virus (Figure 2B).

Figure 2
*Anemarrhena asphodeloides* polysaccharide had no effect on cells, PEDV activation, and PEDV release. (A) Vero cells were pretreated with varying concentrations of *Anemarrhena asphodeloides* polysaccharide (100, 200, and 500 µg/mL) and were subsequently infected with PEDV HLJBY (MOI = 0.1). At 24 hpi, the levels of PEDV N protein were assessed using the Western blot. (B) PEDV HLJBY (MOI = 0.1) was pretreated with *Anemarrhena asphodeloides* polysaccharide at concentrations of 100, 200, and 500 µg/mL before being introduced to Vero cells. Cell samples were collected at 24 hpi and the expression of *β*‐actin and PEDV N proteins was analyzed by Western blot. (C) To assess the effects of *Anemarrhena asphodeloides* polysaccharide on PEDV release, Vero cells were initially infected with PEDV HLJBY at 0.1 MOI at 37°C for 1 h, followed by incubation with different concentrations of *Anemarrhena asphodeloides* polysaccharide. At 24 hpi, TCID_50_ was employed to determine the ratio of virus titer in the cell supernatant to the virus titer in the cells. The error bars represent the standard deviations measured in three separate experiments.

**Figure:**
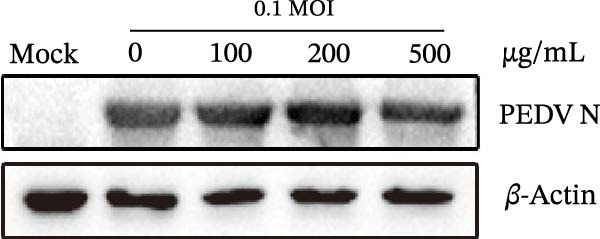
(A)

**Figure:**
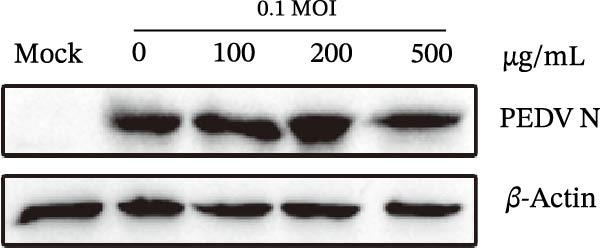
(B)

**Figure:**
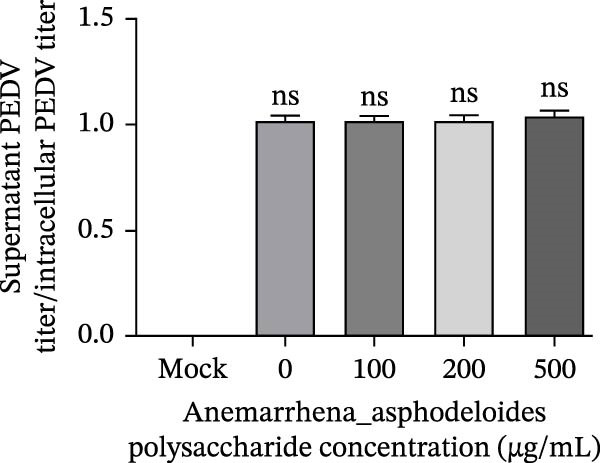
(C)

### 3.3. Effects of *Anemarrhena asphodeloides* Polysaccharide on PEDV Release

To assess the potential impact of polysaccharide treatment on viral release, Vero cells were initially infected with PEDV HLJBY (0.1 MOI) at 37°C for 1 h before the addition of *Anemarrhena asphodeloides* polysaccharide. The PEDV titer was determined by collecting both the supernatant and intact cells. The ratio of PEDV titer in the supernatant to PEDV titer in the cells revealed that treatment with *Anemarrhena asphodeloides* polysaccharide had no discernible effect on PEDV release (Figure 2C).

### 3.4. Targets of *Anemarrhena asphodeloides* Polysaccharide and PEDV

To further investigate the mechanism of *Anemarrhena asphodeloides* polysaccharide disrupting PEDV infection, the Venn diagram drawn by an online platform displayed 35 common targets of *Anemarrhena asphodeloides* polysaccharide and PEDV (Figure 3A). To delve deeper into potential PPIs among these 35 targets, PPI analysis was conducted. Upon uploading this gene data to the STRING database, the PPI network was generated, which consisted of 40 nodes, 253 connections, and an average node degree of 12.7 (Figure 3B). These targets are considered key proteins that *Anemarrhena asphodeloides* polysaccharide exerted antiviral activity. The targets of *Anemarrhena asphodeloides* polysaccharide and PEDV were visualized by Cytoscape software in the form of drug‐targets‐disease network. The 35 common targets were shown in orange, and other targets were shown in blue (Figure 3C).

Figure 3Network establishment and analysis. (A) Venn diagram of the common targets between of *Anemarrhena asphodeloides* polysaccharide and PEDV. (B) Protein–protein interaction (PPI) network analysis. (C) Drug‐target‐disease visualization network. (D–F) GO function. (G) KEGG pathway enrichment analysis. (H) Drug&disease‐targets‐pathways network analysis.

**Figure:**
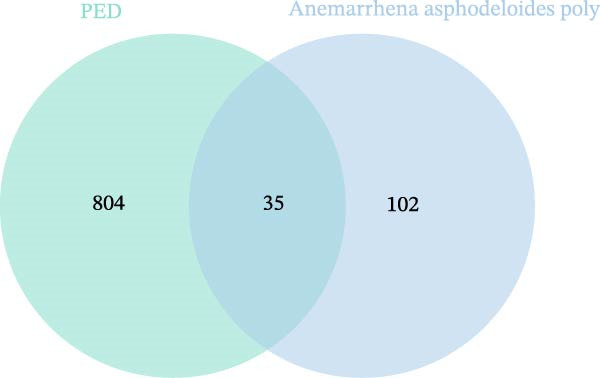
(A)

**Figure:**
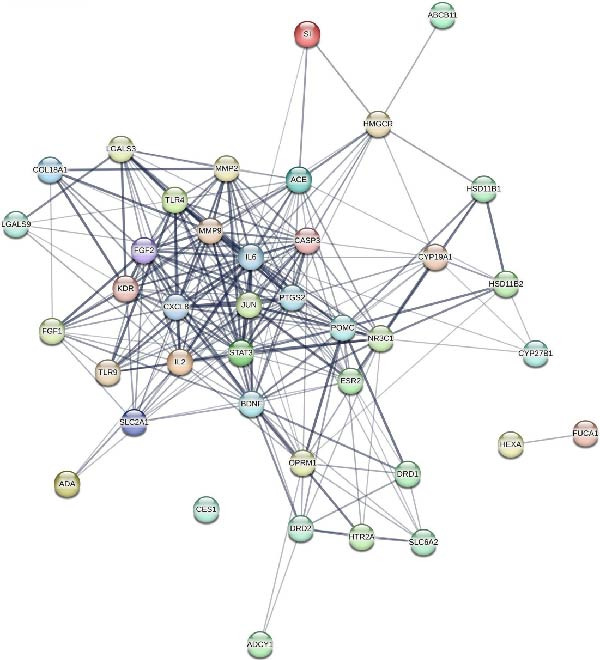
(B)

**Figure:**
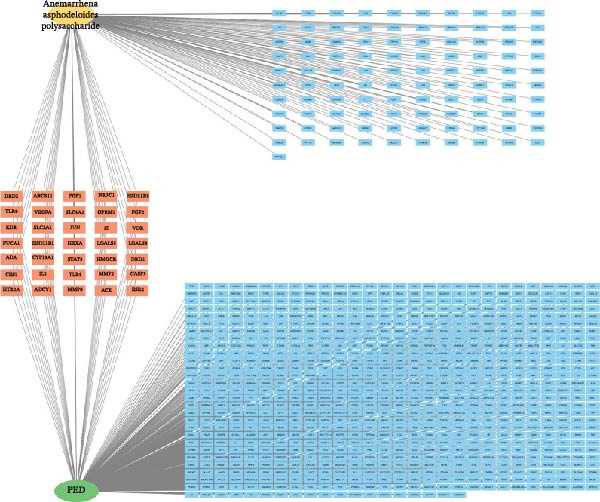
(C)

**Figure:**
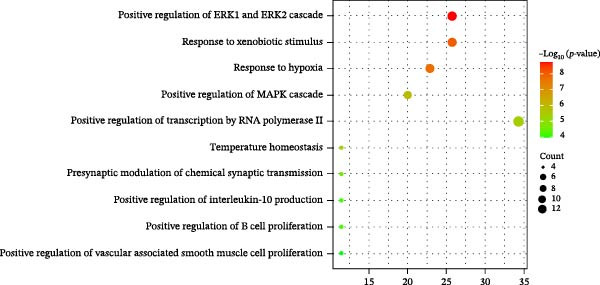
(D)

**Figure:**
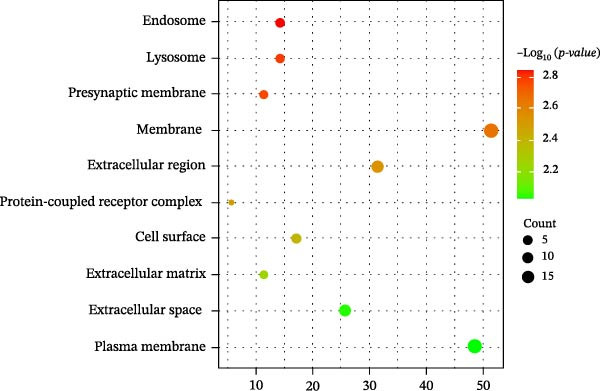
(E)

**Figure:**
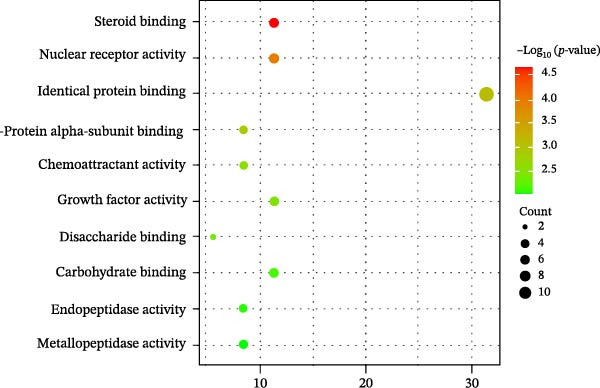
(F)

**Figure:**
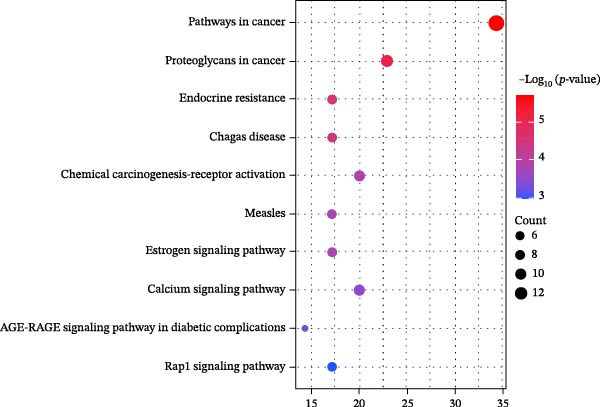
(G)

**Figure:**
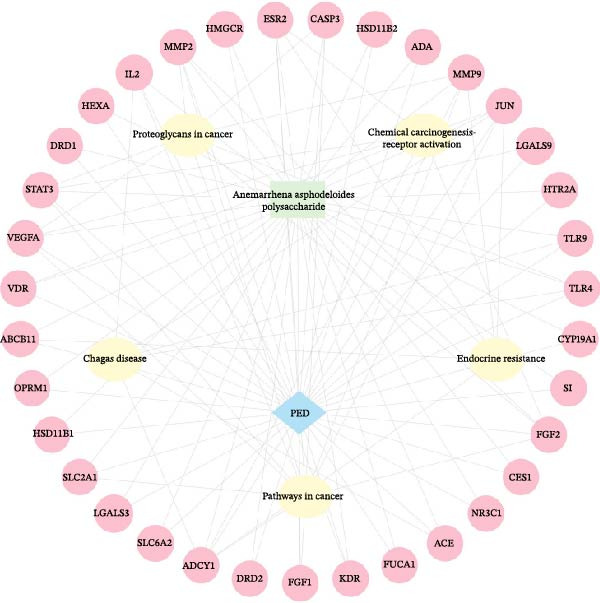
(H)

### 3.5. Network Construction and Analysis of Target Genes

The DAVID online website was used to analyze the GO function and KEGG pathway enrichment data of 35 common targets of *Anemarrhena asphodeloides* polysaccharide and PEDV. Figure 3D–F showed the top 10 BP, CC, and MF of the 35 common targets. The regulation of interleukin‐10 production, ERK1 and ERK2 cascade, and MAPK cascade were vital in biological processes of the targets. The main cellular components were multiple organelles and receptor complex. Besides, the molecular functions of the targets were mainly concentrated on steroid binding and chemoattractant activity. At the same time, 50 pathways were obtained using KEGG enrichment analysis. Figure 3G showed the top 10 pathways, including pathways in cancer and endocrine resistance, etc. The common targets of *Anemarrhena asphodeloides* polysaccharide and PEDV and the KEGG enrichment analysis data were imported into the Cytoscape to establish one Drug&Disease–Targets‐Pathways network (Figure 3H).

### 3.6. *Anemarrhena asphodeloides* Polysaccharide Disrupts Apoptosis to Reduce PEDV Infection

According to the network pharmacology and bioinformatics analysis, ERK1 and ERK2 cascade and MAPK cascade were vital in BP of the targets, and the signal pathways of KEGG enrichment analysis were endocrine resistance. H_2_O_2_ induced ERK1/2 activation, and oxidative stress‐meditated apoptosis is mediated by ERK1/2 phosphorylation [17]. Estrogen deprivation can induce the activation of the caspase cascade, leading to apoptosis [18]. Cell apoptosis is the characteristic of cell lysis caused by viral infections, leading to cytopathic effects (CPE) in vitro and is also one of the causes of viral diseases in vivo, resulting in cell damage, tissue injury, and increased disease severity [19–21]. Therefore, we focused on the effect of *Anemarrhena asphodeloides* polysaccharide on apoptosis.

When Vero cells reached about 70% confluence, the cells were pretreated with different concentrations of *Anemarrhena asphodeloides* polysaccharide (100, 200, and 500 μg/mL) in 2% DMEM and infected with PEDV HLJBY (MOI = 0.1). The cells were infected for 1 h. Then, add *Anemarrhena asphodeloides* polysaccharide (100, 200, 500 μg/mL) diluted in DMEM with 2% in an incubator at 37°C containing 5% CO_2_. After 24 h, cells were stained with Annexin V‐FITC and propidium iodide (PI) and analyzed by flow cytometry. Flow cytometry results indicated that PEDV infection led to increased late apoptosis and total apoptosis rates in Vero cells (Figure 4A–C). However, after the addition of *Anemarrhena asphodeloides* polysaccharide, these rates of apoptosis were reduced compared to the Vero cells only infected with PEDV (Figure 4A–C). Western blot analysis revealed that the level of cleaved caspase‐3 protein increased following PEDV infection, while the addition of *Anemarrhena asphodeloides* polysaccharide led to the reduction in cleaved caspase‐3 protein level (Figure 4D,E). These findings suggest that *Anemarrhena asphodeloides* polysaccharide can disrupt caspase‐3 apoptosis induced by PEDV infection.

Figure 4
*Anemarrhena asphodeloides* polysaccharose reduces apoptosis to inhibit PEDV infection. (A‐D)Vero cells were pretreated with different concentrations of *Anemarrhena asphodeloides* polysaccharide for 1 h, then infected with PEDV HLJBY with MOI = 0.1 for 1 h, then DMEM containing 2% FBS was changed and the corresponding concentration of *Anemarrhena asphodeloides* polysaccharide was added for 24 h. (A) Cells were collected and apoptosis was quantified by Annexin V and PI staining. Fluorescence was analyzed by flow cytometry. (B) Total apoptosis rate, and (C) late apoptosis rate were detected. Early apoptotic cells (Annexin V positive). Late apoptotic cells (Annexin V and PI positive). Early apoptosis plus late apoptosis equals total apoptosis. (D) Western blotting detected changes in cleaved caspase‐3 protein concentration. (E) Density intensity of banded cleaved caspase‐3 was measured and normalized according to *β*‐actin expression. (F–I) Vero cells were pretreated with different concentrations of Ac‐DEVD‐CHO for 1 h, then infected with PEDV HLJBY with MOI = 0.1 for 1 h, then DMEM containing 2% FBS was changed and the corresponding concentration of Ac‐DEVD‐CHO was added for 24 h. (F) The cells were collected for Western blotting to detect changes in cleaved caspase‐3 protein and PEDV N protein concentration. (G and H) Density intensity of banded cleaved caspase‐3 and PEDV‐N was measured and normalized according to *β*‐actin expression. (I) Virus titers were measured by TCID_50_. ns, no significant;  ^∗^ *p* < 0.05,  ^∗∗^ *p* < 0.01.

**Figure:**
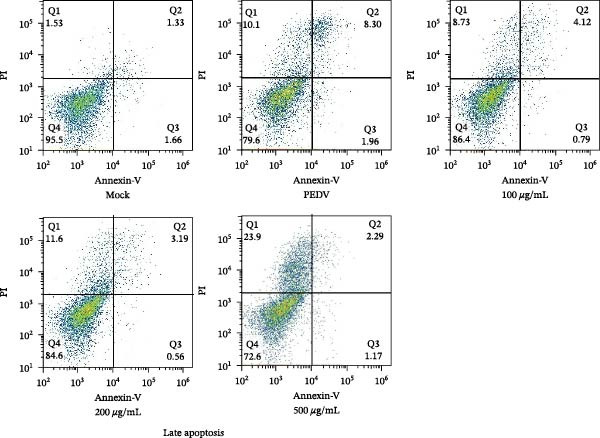
(A)

**Figure:**
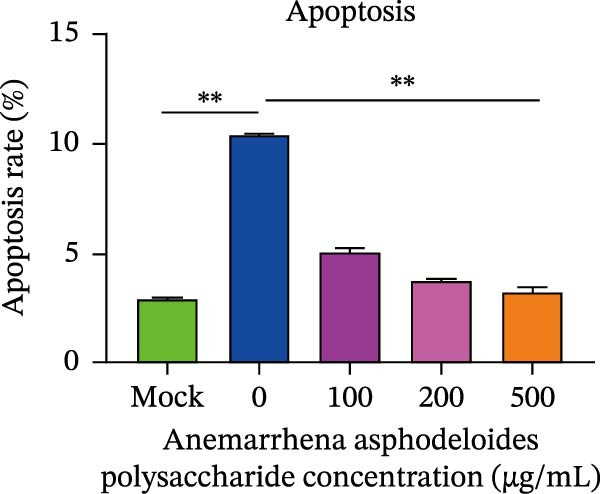
(B)

**Figure:**
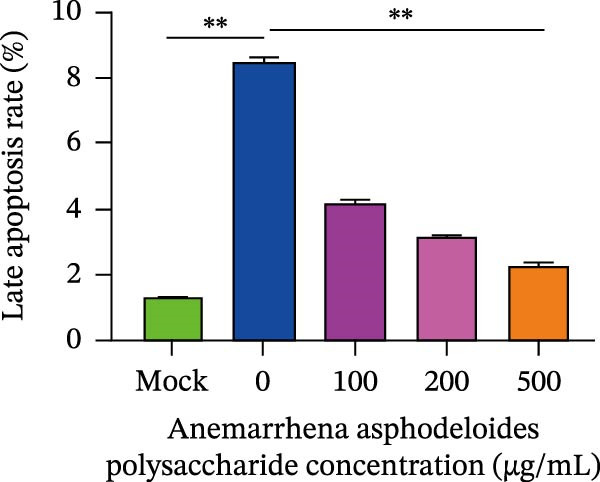
(C)

**Figure:**
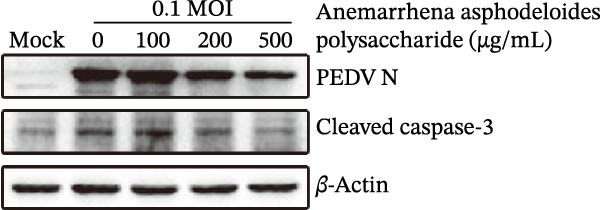
(D)

**Figure:**
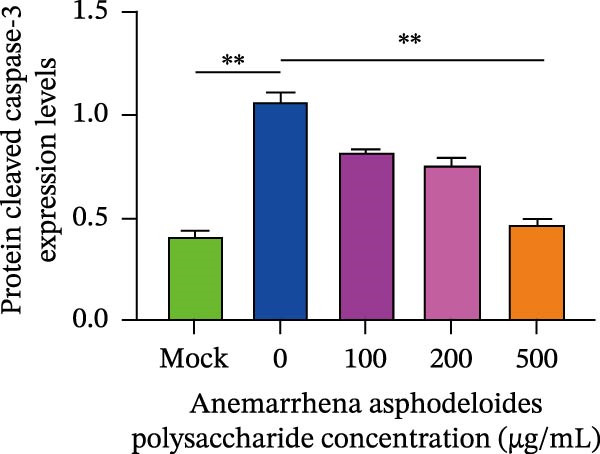
(E)

**Figure:**
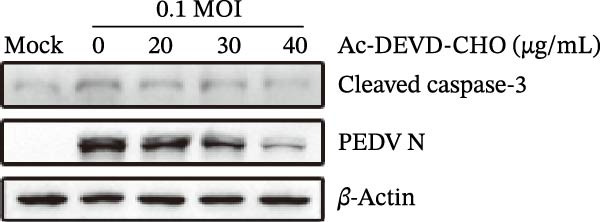
(F)

**Figure:**
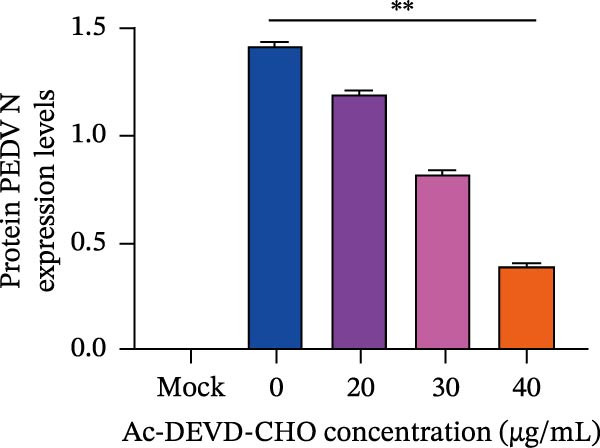
(G)

**Figure:**
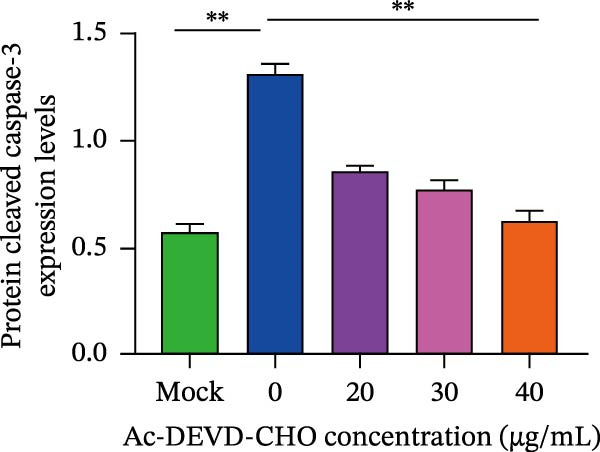
(H)

**Figure:**
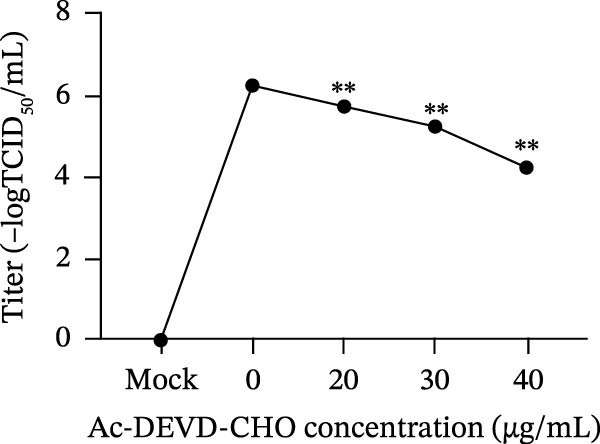
(I)

To explore whether *Anemarrhena asphodeloides* polysaccharide targets caspase‐3 depended apoptosis to inhibit PEDV infection, we used caspase‐3 inhibitor Ac‐DEVD‐CHO to treat Vero cells infected PEDV. Western blot revealed that Ac‐DEVD‐CHO inhibited the level of cleaved caspase‐3 protein and PEDV N protein (Figure 4F–H). TCID_50_ demonstrated that Ac‐DEVD‐CHO reduced the titer of PEDV, and 40 μM Ac‐DEVD‐CHO reduced the viral titer by ~100 times compared to the mock condition (Figure 4I). These results suggested that caspase‐3 inhibitor Ac‐DEVD‐CHO inhibited PEDV infection.

### 3.7. *Anemarrhena asphodeloides* Polysaccharose Reduces Caspase‐3 Depended Apoptosis by Targeting ROS to Suppress PEDV Infection

After viruses invade host, they promote the generation of oxidants and inhibit the activity of related antioxidant enzymes. In this way, viruses disrupt the original dynamic equilibrium within the host’s cells, thereby causing the host to enter an oxidative stress state. When an organism is in the state of oxidative stress, it inhibits cells from taking antiviral actions, thereby to some extent increasing the risk of viral infection [22]. Increased ROS production in the cell leads to the activation of ERK, JNK, or p38 MAPKs [23]. Estrogen deprivation can significantly increase the expression of oxidative stress response genes [24]. H_2_O_2_ treatment can cause the production of ROS in porcine follicular cells and trigger cell apoptosis [25]. Acetylcysteine (NAC) significantly attenuated mitochondrial dysfunction and apoptosis induced by glucose oxidative stress [26]. Oxidative stress and apoptosis are closely linked physiological phenomena and the chronic diseases including cancer, AIDS, diabetes mellitus, and autoimmunity [27]. Therefore, we explored that the role of *Anemarrhena asphodeloides* polysaccharide on oxidative stress. Vero cells were infected with PEDV HLJBY (MOI = 0.1) with different concentrations of *Anemarrhena asphodeloides* polysaccharide (100, 200, 500 μg/mL) and incubated at 37°C for 24 h. The levels of intracellular ROS were quantified using DCFH‐DA. The results are depicted in Figure 5A,B, illustrating the increase of ROS induced by PEDV infecion, and significant reduction in intracellular ROS levels reduced by *Anemarrhena asphodeloides* polysaccharide treatment. In addition, the activity of superoxide dismutase (SOD), the content of malondialdehyde (MDA), and glutathione peroxidase (GSH‐px) activity were measured via the kit. Experimental results demonstrated that PEDV infection significantly elevated intracellular MDA levels (Figure 5C), reduced SOD content (Figure 5D), and decreased GSH‐px activity in Vero cells (Figure 5E). When processed with *Anemarrhena asphodeloides* polysaccharide, the Vero cells showed progressive decreases in MDA (Figure 5D), a gradual recovery of SOD (Figure 5C), and increased GSH‐px activity (Figure 5E). These findings indicate that PEDV infection triggers substantial ROS production, inducing oxidative stress and compromising cellular antioxidant capacity. Excessive ROS induce lipid peroxidation, leading to significant MDA accumulation. Notably, *Anemarrhena asphodeloides* polysaccharide effectively enhances cellular antioxidant responses, thereby mitigating both oxidative stress and lipid peroxidation caused by PEDV infection.

Figure 5
*Anemarrhena asphodeloides* polysaccharose reduces caspase‐3 depended apoptosis by downregulating ROS to disrupt PEDV infection. We pretreated Vero cells with different doses of *Anemarrhena asphodeloides* polysaccharide for 1 h (MOI = 0.1) before PEDV HLJBY infection, and then treated with different doses of *Anemarrhena asphodeloides* polysaccharide for 24 h. (A) Flow cytometry measures the total fluorescence intensity of more than 10,000 cells in each sample. (B) The levels of ROS were quantified by fluorescence density. (C) The cells were lyzed, and the supernant was taken according to the kit instructions to detect the intracellular SOD levels. (D) The cells were lyzed, and the supernatant was taken according to the kit instructions to detect intracellular MDA levels. (E) The cells were lyzed, and the supernant was taken according to the kit instructions to detect the intracellular GSH‐Px levels. (F) Cells were collected and apoptosis was quantified by Annexin V and PI staining. Fluorescence was analyzed by flow cytometry. (G) Total apoptosis rate, and (H) late apoptosis rate were detected. Early apoptotic cells (Annexin V positive), Late apoptotic cells (Annexin V and PI positive). Early apoptosis plus late apoptosis equals total apoptosis. (I) Western blotting detected changes in cleaved caspase‐3 and PEDV ‐N protein concentration. (J and K) Density intensity of banded cleaved PEDV ‐N and caspase‐3 was measured and normalized according to *β*‐actin expression. (L) Virus titers were measured by TCID_50_.  ^∗^ *p* < 0.05,  ^∗∗^ *p* < 0.01.

**Figure:**
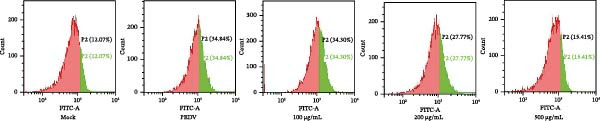
(A)

**Figure:**
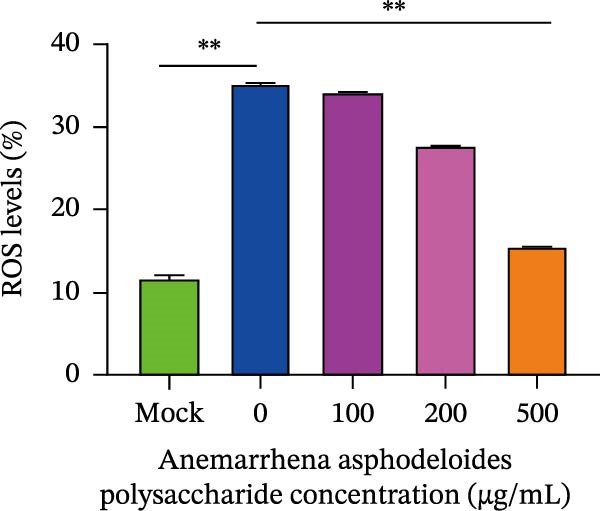
(B)

**Figure:**
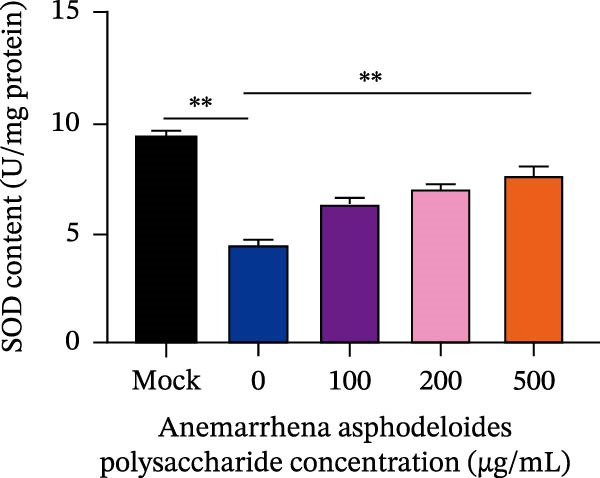
(C)

**Figure:**
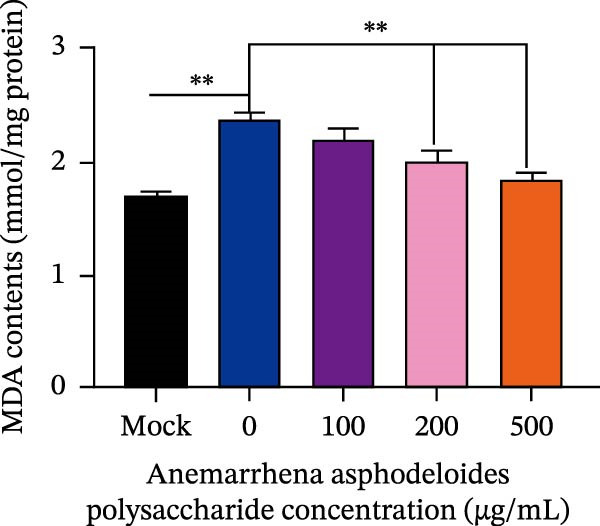
(D)

**Figure:**
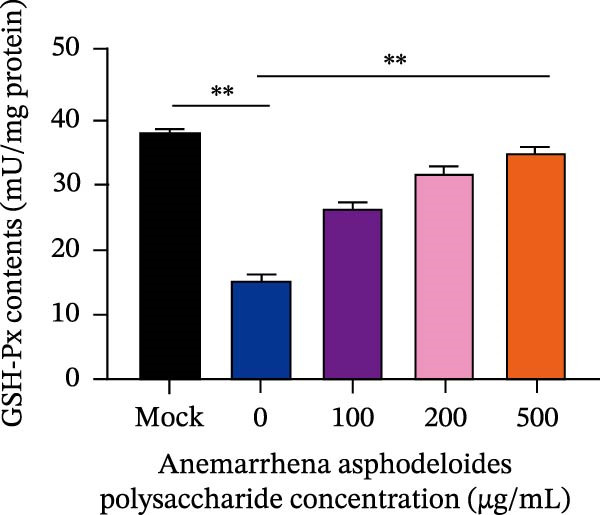
(E)

**Figure:**
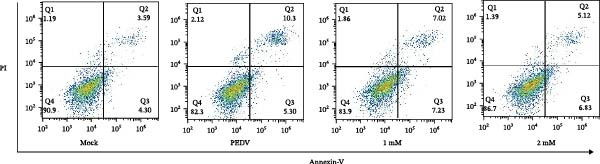
(F)

**Figure:**
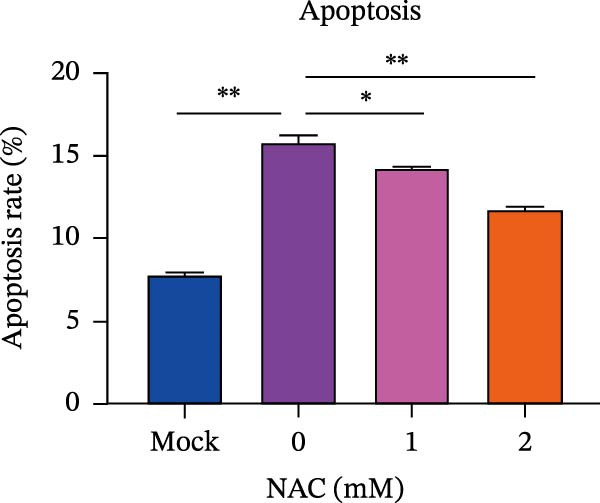
(G)

**Figure:**
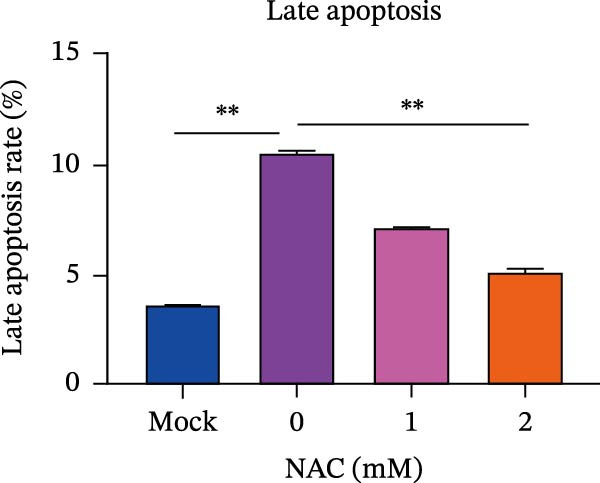
(H)

**Figure:**
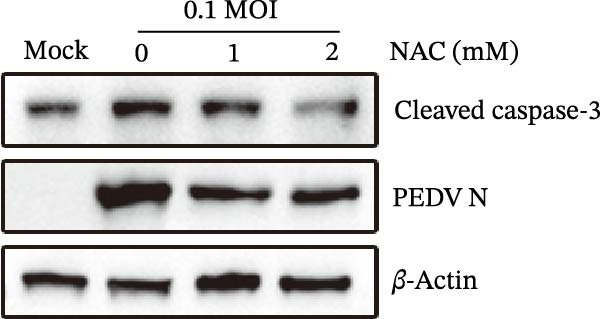
(I)

**Figure:**
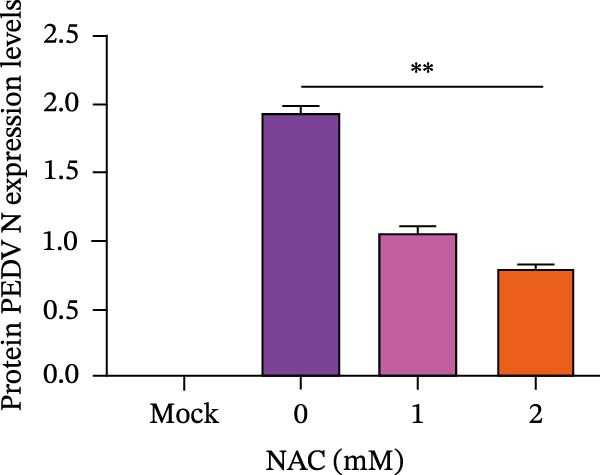
(J)

**Figure:**
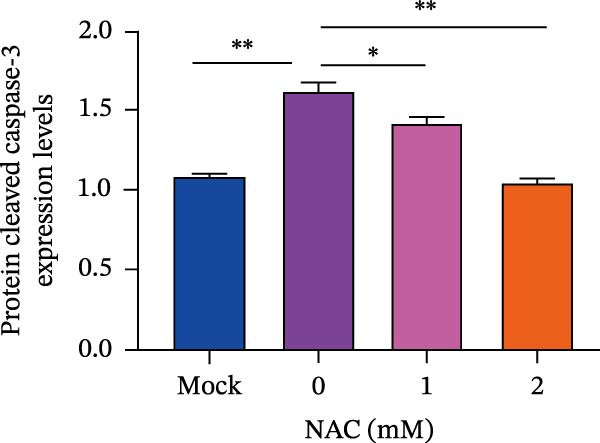
(K)

**Figure:**
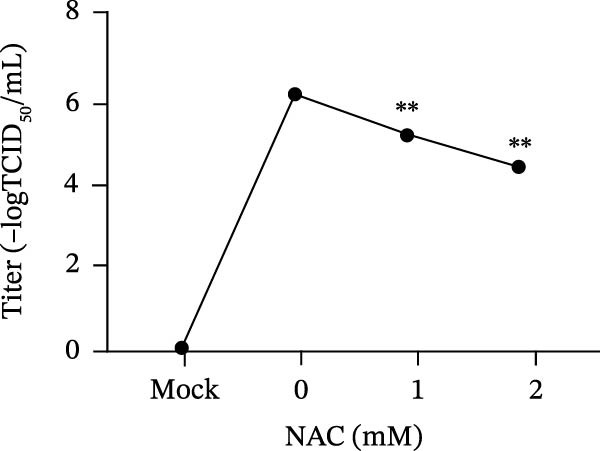
(L)

To determine whether *Anemarrhena asphodeloides* polysaccharide reduced caspase‐3 depended apoptosis by targeting ROS to inhibit PEDV infection, we used ROS scavenger NAC (1, 2 mM) to treat Vero cells infected with PEDV. Flow cytometry results indicated that ROS scavenger NAC led to the decrease in late apoptosis and total apoptosis rates (Figure 5F–H). Western blot demonstrated that the reduction in PEDV N protein following NAC treatment, as well as the decrease in cleaved caspase‐3 protein levels (Figure 5I–K). TCID_50_ demonstrated that NAC reduced the titer of PEDV, and 2 mM NAC reduced the viral titer by ~60 times compared to the mock condition (Figure 5L). These results approved that ROS can induce caspase‐3 depended apoptosis to increase PEDV infection.

**Figure 6:**
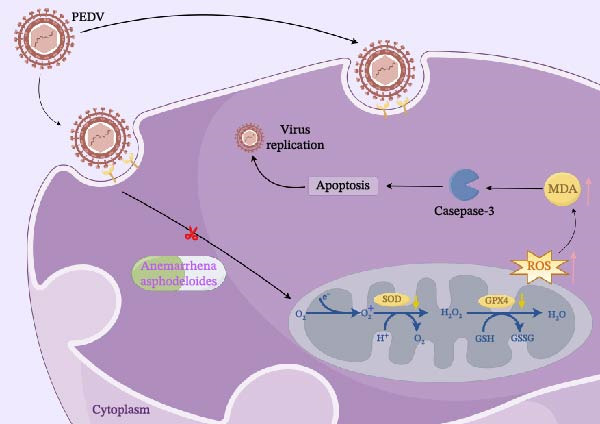
Illustration of the mechanism underlying the antiviral role of *Anemarrhena asphodeloides* polysaccharide against PEDV.

In conclusion, we reveal the antiviral mechanism of *Anemarrhena asphodeloides* polysaccharide against PEDV (Figure 6).

## 4. Discussion

In recent decades, PEDV has emerged as a significant threat to the pig industry, resulting in substantial economic losses, and effective vaccines are still limited [28]. Consequently, the development of novel anti‐PEDV agents holds paramount importance. Plant polysaccharides, which are abundant in plant roots, stems, and leaves, have garnered attention as promising biomacromolecules [29, 30]. These compounds have been the subject of numerous studies due to their reported antiviral effects, with the added advantage of being generally nontoxic and causing minimal side effects [31]. The spectrum of activities exhibited by plant polysaccharides includes immunomodulation, antitumor properties, radiation protection, and antiviral effects [32–34]. For instance, *Polygonum cillinerve* polysaccharide has demonstrated its antiviral capabilities by impeding the replication of transmissible gastroenteritis virus (TGEV) while reducing TGEV‐induced apoptosis and levels of ROS [35]. Similarly, *Panax Notoginseng* polysaccharide exerts its antiviral effects primarily by inhibiting the adsorption and replication of PRV in vitro [36]. Consequently, plant polysaccharides hold promise as potential source for the development of antiviral drugs.

*Anemarrhena asphodeloides* polysaccharide is isolated from the rhizome of *Anemarrhena asphodeloides* and has been previously known for its blood sugar‐lowering effects [37]. Furthermore, it has been observed that *Anemarrhena asphodeloides* polysaccharides can stimulate RAW264.7 cells to produce significant amounts of NO and upregulate the expression of TNF‐α, IL‐1, and COX‐2 genes. Additionally, it has exhibited growth‐inhibitory effects on cancer cell lines such as AGS, MKN‐28, and MKN‐45 [10]. However, there have not been any prior studies reporting the inhibitory effect of this polysaccharide on PEDV. The results show that *Anemarrhena asphodeloides* polysaccharide indeed has an inhibitory effect on PEDV infection. However, the infectivity of PEDV was not affected by cells pretreated with *Anemarrhena asphodeloides* polysaccharide. The deactivation and release of PEDV were not affected by the *Anemarrhena asphodeloides* polysaccharide. Therefore, it has been established that *Anemarrhena asphodeloides* polysaccharide can significantly inhibit PEDV HLJBY infection in vitro.

According to the network pharmacology and bioinformatics analysis, ERK1 and ERK2 cascade and MAPK cascade were vital in biological processes of the targets, and the signal pathways of KEGG enrichment analysis were endocrine resistance. ERK1, ERK2 cascade, MAPK cascade, endocrine resistance, and oxidative stress had close relationship. ROS are known to be toxic byproducts of cellular metabolism and play a role in oxidative stress during various viral infections [38]. Under normal conditions, cells maintain the balance of ROS through antioxidant systems [39]. However, under stress conditions such as ionizing radiation, chemotherapy drugs, and viral infections, ROS levels can accumulate excessively and influence various pathological processes such as proliferation, inflammation, autophagy, and apoptosis [40]. Several studies have indicated that ROS are involved in virus‐induced apoptosis. Previous studies have reported that plant polysaccharides possess antioxidant properties and can reduce ROS levels [41, 42]. PEDV infection and cellular apoptosis exhibit the reciprocal regulatory relationship, wherein PEDV possesses the ability to both induce and inhibit apoptotic processes. Cellular apoptosis assumes a dual function in the context of PEDV replication. It serves as the host defense mechanism aimed at restricting viral replication and dissemination. Conversely, certain viruses have developed strategies to exploit apoptotic pathways to enhance their own replication. Our results reveal that PEDV infection induce caspase‐3 depended on apoptosis, and intracellular ROS levels in Vero cells increased after PEDV infection. In addition, caspase‐3 dependent on apoptosis and ROS are significantly decreased after treatment with *Anemarrhena asphodeloides* polysaccharide. Apoptosis inhibitor Ac‐DEVD‐CHO and ROS scavenger NAC disrupt PEDV infection, and NAC reduces caspase‐3 dependent apoptosis. This study provides preliminary evidence of the anti‐PEDV activity of *Anemarrhena asphodeloides* polysaccharide by targeting ROS/caspase‐3 dependent apoptosis.

In summary, our study provides novel insights into the anti‐PEDV activity of *Anemarrhena asphodeloides* polysaccharide. *Anemarrhena asphodeloides* polysaccharide has been shown to inhibit PEDV infection in dose‐dependent manner. Furthermore, *Anemarrhena asphodeloides* polysaccharide significantly reduces intracellular ROS levels and caspase‐3 dependent apoptosis in infected cells. Therefore, *Anemarrhena asphodeloides* polysaccharide against PEDV infection targeting ROS/caspase‐3 dependent apoptosis.

## Funding

This research was funded by the National Natural Science Foundation of China (Grant 32273015), a project funded by the Priority Academic Program Development of Jiangsu Higher Education Institutions (PAPD), and supported by the 111 Project (Grant GD18007) and the Top‐level Talents Support Program of Yangzhou University.

## Conflicts of Interest

The authors declare no conflicts of interest.

## Data Availability

The potential targets of PEDV were obtained from GeneCards (https://www.genecards.org/). Subsequently, the Canonical SMILES expressions of these identified D‐mannose, L‐rhamnose, D‐galacturonic acid, D‐glucose, D‐galactose, and L‐arabinose were searched in the PubChem database (https://www.pubchem.ncbi.nlm.nih.gov/). Then, based on the two‐dimensional and three‐dimensional characteristics of the known ligand, potential molecular targets were predicted using the Swiss Target Prediction database (http://www.swisstargetprediction.ch/).
